# Metagenomic analysis of the medicinal leech gut microbiota

**DOI:** 10.3389/fmicb.2014.00151

**Published:** 2014-04-17

**Authors:** Michele A. Maltz, Lindsey Bomar, Pascal Lapierre, Hilary G. Morrison, Emily Ann McClure, Mitchell L. Sogin, Joerg Graf

**Affiliations:** ^1^Department of Molecular and Cell Biology, University of ConnecticutStorrs, CT, USA; ^2^Marine Biological Laboratory, The Josephine Bay Paul CenterWoods Hole, MA, USA

**Keywords:** high-throughput sequencing, beneficial microbes, symbiosis, medicinal leech

## Abstract

There are trillions of microbes found throughout the human body and they exceed the number of eukaryotic cells by 10-fold. Metagenomic studies have revealed that the majority of these microbes are found within the gut, playing an important role in the host's digestion and nutrition. The complexity of the animal digestive tract, unculturable microbes, and the lack of genetic tools for most culturable microbes make it challenging to explore the nature of these microbial interactions within this niche. The medicinal leech, *Hirudo verbana*, has been shown to be a useful tool in overcoming these challenges, due to the simplicity of the microbiome and the availability of genetic tools for one of the two dominant gut symbionts, *Aeromonas veronii*. In this study, we utilize 16S rRNA gene pyrosequencing to further explore the microbial composition of the leech digestive tract, confirming the dominance of two taxa, the *Rikenella*-like bacterium and *A*. *veronii*. The deep sequencing approach revealed the presence of additional members of the microbial community that suggests the presence of a moderately complex microbial community with a richness of 36 taxa. The presence of a *Proteus* strain as a newly identified resident in the leech crop was confirmed using fluorescence *in situ* hybridization (FISH). The metagenome of this community was also pyrosequenced and the contigs were binned into the following taxonomic groups: *Rikenella*-like (3.1 MB), *Aeromonas* (4.5 MB), *Proteus* (2.9 MB), *Clostridium* (1.8 MB), *Eryspelothrix* (0.96 MB), *Desulfovibrio* (0.14 MB), and *Fusobacterium* (0.27 MB)**. Functional analyses on the leech gut symbionts were explored using the metagenomic data and MG-RAST. A comparison of the COG and KEGG categories of the leech gut metagenome to that of other animal digestive-tract microbiomes revealed that the leech digestive tract had a similar metabolic potential to the human digestive tract, supporting the usefulness of this system as a model for studying digestive-tract microbiomes. This study lays the foundation for more detailed metatranscriptomic studies and the investigation of symbiont population dynamics.

## Introduction

Microbes residing within and on the human body are estimated to exceed the number of host eukaryotic cells 10-fold. The majority of these microbes are harbored in the gut (Qin et al., 2010), where they have been shown to aid in the digestion of food and the provision of essential nutrients to the host (Ley et al., 2006; Xu et al., 2007; Turnbaugh et al., 2009). As most microbes are not culturable under laboratory conditions, culture-independent approaches are necessary to gain further knowledge about the roles these symbionts perform inside their host. One such culture-independent technique is metagenomics, in which genomes of a microbial community are sequenced, thereby revealing the metabolic potential of the community. Advances in massively parallel sequencing have opened up new approaches for studying digestive-tract microbiomes, proving to be important tools for exploring complex microbial environments. The complexity of the human gut community can encompass hundreds of operational taxonomic units (OTUs) (Human Microbiome Project, 2012a,b). This complexity of the human gut microbiome, sampling depth constraints, and large amounts of sequence data pose challenges for studying digestive-tract microbiota. Available versatile invertebrate models with simpler microbial communities may overcome these challenges and provide important new insights into gut microbial symbioses (Ruby, 2008).

**Figure 1:**
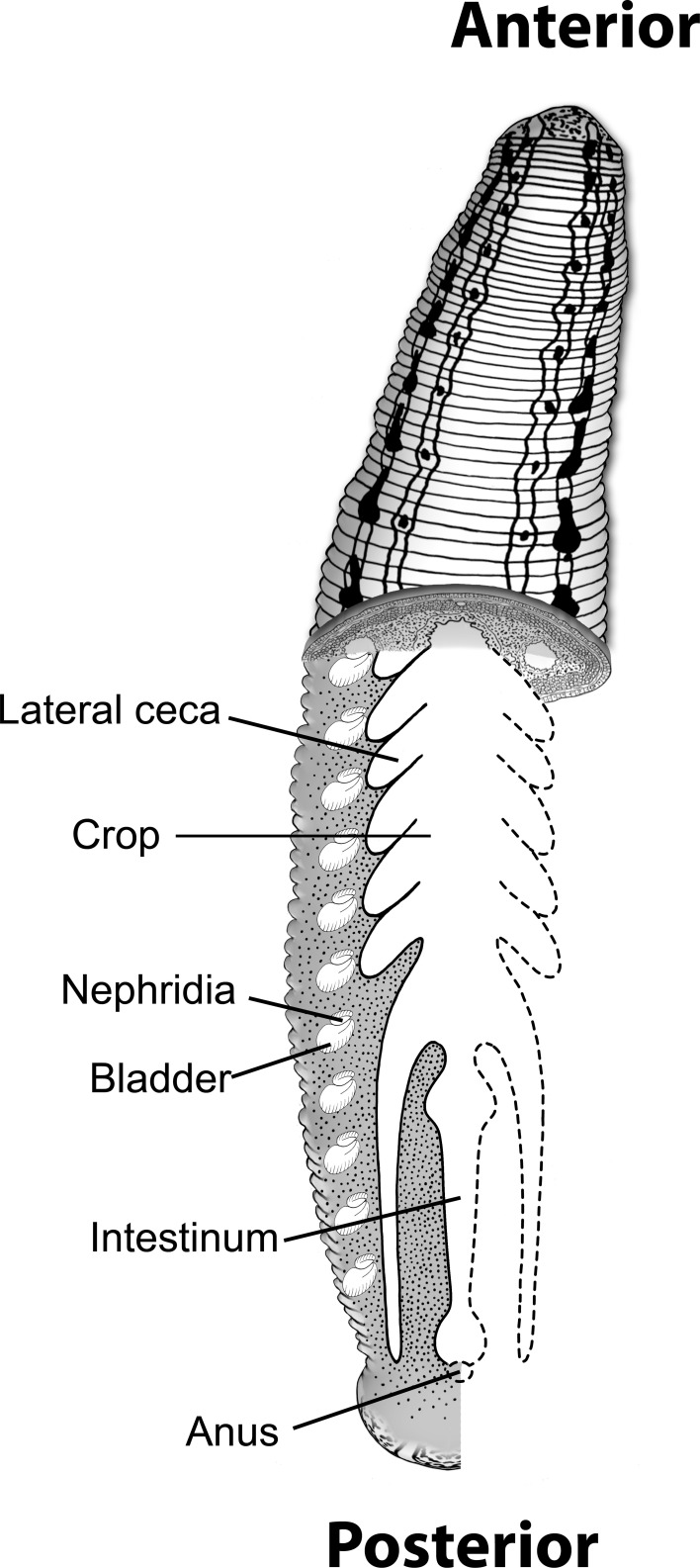
**Model of leech digestive tract**. Image depicts the different parts of the leech digestive tract (modified from Nelson and Graf, 2012). The ingested blood meal is stored in the crop and most of the actual digestion is thought to occur in the intestinum.

The medicinal leech, *Hirudo verbana*, has been shown to be a versatile invertebrate model system for digestive-tract symbioses with powerful molecular tools (Graf et al., 2006; Ruby, 2008; Nelson and Graf, 2012; Nyholm and Graf, 2012). The leech digestive tract is comprised of two major compartments: the crop, the largest region where the ingested blood meal is stored, and the intestinum, a much smaller region where the blood meal is digested (Kikuchi and Graf, 2007; Laufer et al., 2008) (Figure 1). In a single feeding, the medicinal leech can consume over five times its body weight in blood. Inside the leech crop, water and salts are discharged from the blood meal, to produce a highly viscous intraluminal fluid (ILF). The ILF contains densely packed, partially lysed, erythrocytes (from the meal), and host macrophage-like cells called hemocytes that phagocytose sensitive bacteria (Kikuchi and Graf, 2007; Silver et al., 2007).

Previous studies on the microbial composition of the crop using both culture-dependent and -independent approaches, revealed a very simple microbiome in the crop that is dominated by two species: *Aeromonas veronii* and a *Rikenella*-like bacterium. *A. veronii* is a Gram-negative, facultative anaerobe that has been shown to lyse erythrocytes present in the crop (Maltz and Graf, 2011) and is resistant to hemocyte phagocytosis (Kikuchi and Graf, 2007; Silver et al., 2007). The *Rikenella*-like bacterium is a member of the Bacteroidetes and likely represents a novel genus related to *Rikenella microfusus* (Kaneuchi and Mitsuoka, 1978; Worthen et al., 2006), a species isolated from cecal and fecal samples of Japanese calves, chickens, and quails (Kaneuchi and Mitsuoka, 1978). These two species account for ≥97% of the 16S rRNA gene sequences analyzed (Graf, 1999; Worthen et al., 2006; Kikuchi and Graf, 2007). The same studies also suggest a greater diversity of microorganisms in the intestinum due to the presence of additional low abundance organisms (Graf, 1999; Worthen et al., 2006; Laufer et al., 2008; Bomar et al., 2011). Also within the crop, three different microbial habitats involving both of these dominant microbes have been identified; host epithelium-associated, free-living and microcolony-associated bacteria (Kikuchi and Graf, 2007).

There are three main hypotheses as to why these symbiotic relationships are maintained. The first hypothesis is that these gut microbes aid in the digestion of the blood meal (Hornborstel, 1942; Büsing, 1951). Previous studies have shown that *Aeromonas* possess a hemolysin that lyses erythrocytes in the crop (Bomar et al., 2011). The second hypothesis is that the gut symbionts provide the leech with essential nutrients that cannot be obtained from the diet (Edward et al., 1957; Graf, 2002). A recent metatranscriptome study on the leech crop revealed that the dominant symbionts may be fermenting sugars and releasing acetate which could be used as a nutrient source by the host (Bomar and Graf, 2012). The third hypothesis is that these symbionts play a role in the protection of the gut against colonization by pathogenic bacteria (Büsing et al., 1953). In the study done by Indergand and Graf, pathogenic bacteria were introduced and unable to colonize the crop suggesting that the community is either tightly controlled by the leech host and/or the microbial community creates conditions unsuitable for these microbes to survive (Indergand and Graf, 2000). Further experimental investigation is needed to test each of these hypotheses; the long life span of leeches makes these important functional analyses more challenging. Metagenomic studies lay the foundation for addressing these hypotheses of not only the dominant microbes in this symbiotic relationship, but also the less abundant members. Metagenomics has the potential to reveal the presence of different metabolic pathways and/or genes important for scavenging nutrients, which can lead to a better understanding of the likely function performed by all of the microbes involved in this symbiotic relationship.

Genetic information from a microbial community can lead to testable hypotheses about the function of the microbiome even when complete enzymatic pathways are not always performed by a single organism or in coordination with the host (Wilson et al., 2010; Burnum et al., 2011). In mixed-species microcolonies, close proximity necessitates competition for resources while opening the potential for complementary metabolic processes occurring between species. Division of metabolic labor, as through complementary metabolic processes, or syntrophy, may offer a fitness advantage to cooperating organisms (Morris et al., 2013; Pande et al., 2013; Molloy, 2014). Potentially, interacting organisms can be identified by localization using fluorescent *in situ* hybridization (FISH), a method in which specific nucleotide probes bound to fluorophores are used to identify and localize specific microorganisms within a colony, community or tissue (Ishii et al., 2004; Collins et al., 2012). In conjunction with metagenomic data, FISH analysis supports the metagenomic data, provides information about the abundance of organisms and reveals the location of the microorganisms (Hentschel et al., 2000).

In this study, we used pyrosequencing of 16S rRNA genes to determine the composition of the leech gut microbiome, and revealed the location of a newly discovered inhabitant using fluorescence *in situ* hybridization (FISH) with specific probes. The analysis of the metagenome revealed potential bacterial activities in the host environment and a similarity to other gut metagenomes.

## Materials and methods

### Sampling and DNA extraction

#### For the 16S rRNA gene sequencing

DNA was isolated from the ILF and intestinum of four *H. verbana* specimen 96 h after first feeding heparinized sheep blood (Quad5). A second group of four animals was fed twice, 1 month apart. The animals were purchased from a medical supplier, Leeches USA. All animals were surface sterilized in 70% ethanol prior to dissection of the ILF and intestinum. Samples were stored at −80°C until DNA extraction. Genomic DNA was extracted using MasterPure™ DNA purification kit (Epicentre® Biotechnologies) following the whole blood sample protocol. DNA purity and concentration were analyzed by spectrophotometric quantification and gel electrophoresis. After DNA extraction, equal amounts of DNA were pooled into separated pools for ILF and intestinum.

#### For the shotgun metagenome

DNA isolated from the ILF of *H. verbana* was pooled into one sample from four animals 4 days after the second feeding using blood from which buffy coat containing the leukocytes had been removed by centrifugation.

### Pyrosequencing and sequence analysis

For the 16S rRNA study, the V6 region was amplified as previously described and analyzed using VAMPS (Sogin et al., 2006; Huber et al., 2007; Huse et al., 2007), SRA number PRJNA237098. Using VAMPs, we analyzed the data to the genus level, unless the sequence identification was only available at higher taxonomic level. For the shotgun metagenome, the libraries were prepared from 0.5 to 0.8 kb size-selected, sheared DNA, one library was sequenced as a test run on the 454/Roche GS-FLX using the Titanium chemistry at the Center for Applied Genetic Technology of the University of Connecticut, then the same DNA sample was sent to Genome Quebec at McGill University for library construction and sequencing. The metagenome was then assembled using GS *de novo* Assembler from Roche. The assembly was performed on the combined reads from all sequencing runs (Table 1) SRA Number: SRX337571.

**Table 1:** **Summary of shotgun metagenome data**.

**Sequence run**	**No. of reads used**	**No. of bases used**	**% Reads assembled**	**% Bases assembled**
GS Reader^*^	573,169	223,839,193	90.56	91.63
13 TCA 454^*^	9,755	3,247,229	95.67	95.51
14 TCA 454^*^	2,655	913,881	94.22	93.61
15 TCA 454^*^	21,883	8,256,995	93.48	92.85
16 TCA 454^*^	22,171	8,345,867	92.32	92.85

* *Sequence runs used the same DNA sample that contained a pool of 4 different ILF samples*.

### Contig annotations and binning

Open reading frame (ORF) prediction was performed on contigs of 1000 bp or greater using Glimmer version 3.02 (Delcher et al., 2007) assuming linear fragments and presence of incomplete ORFs. The training dataset for Glimmer prediction was composed of 13 bacterial species that were closely related to members of the metagenome population (*Fusobacterium* sp. 3_1_33, *Fusobacterium nucleatum* subsp. *nucleatum* ATCC 25586, *Flavobacterium johnsoniae* UW101, *Escherichia coli* str. K-12 substr. MG1655, *Clostridium perfringens* str. 13, *Bacteroides vulgatus* ATCC 8482, *Bacteroides fragilis* YCH46, *Bacteroides thetaiotaomicron* VPI-548*, Bacillus subtilis* subsp. *subtilis* str. 168, *Alistipes putredinis* DSM 17216, *Alistipes finegoldii* DSM 17242*, Aeromonas hydrophila* subsp. *hydrophila* ATCC 7966, and *Aeromonas salmonicida salmonicida* A449). The predicted ORFs were compared to the non-redundant GenBank database using Blast2Go (Götz et al., 2008) for annotation purposes and species identification. Contigs were grouped or binned together into organisms bins using Principal Component Analyses (PCA) (Figures 3A,B), K-Mean clustering, and Emergent Self-Organizing Maps (ESOM) (Figure 3C). K-Mean clustering was calculated using normalized GC percent, di-, tri, tetra-, and penta-nucleotide frequencies of each contig in Cluster 3.0 (de Hoon et al., 2004) using 10 clusters and 100 runs with Euclidean distances. The same compositional matrix was used to calculate the Eigenvectors and Eigenvalues for the PCA in Cluster 3.0 using the default settings. Normalized tetra-nucleotide frequencies were used to calculate the self-organizing map with the Databionics ESOM-map software (Ultsch and Moerchen, 2005) using the same parameters as described by Dick et al. (2009). Final results were manually curated for species assignment of contigs based on their similarity in clustering patterns and blast results. Contigs with ambiguous taxonomic assignments (i.e., different assignments from nucleotide usage binning and self-evolving maps) were discarded for the rest of the analysis. Metagenome contigs were also uploaded onto Metagenome Rapid Annotation using Subsystem Technology (MG-RAST, ID number **445547.3)**, and analyzed using the SEED Annotation Engine (http://seed.sdsu.edu/fig/indez.cgi) (Meyer et al., 2008).

### COG and KEGG analysis of different microbiomes

The COG (Clusters of Orthologous Groups) categories for digestive tract and environmental metagenome comparisons (Supplemental Table 2) were determined by comparing the protein sequences to the COG database using BLASTX and an *E*-value cutoff of 10e^−04^. The KEGG (Kyoto Encyclopedia of Genes and Genomes) categories for the same datasets were determined using the KAAS (KEGG Automatic Annotation Server) (Moriya et al., 2007) with the single-directional best hit option over the default representative genome set for prokaryotes. The resulting COG and KEGG data were normalized by dividing the number of hits for each category by the total number of hits. PCA was performed on the normalized data using the XLSTAT software package (Addinsoft, New York, NY).

### Tissue preservation and sectioning

Three leeches were sacrificed 4 daf, relaxed in 70% ethanol (Graf, 1999) and transferred to anhydrous Methyl-Carnoy's (6:3:1 methanol:chloroform:acetic acid). Fixation continued at 4°C, replacing fixative with fresh fixative 6, 12, 18, 24, and 36 h after sacrificing the animals. At 24 h, the leeches were dissected into thirds and the anterior and posterior thirds were discarded. At 48 h, the remaining central section was further dissected into ~2 mm sections. At 48 h, sections were transferred to anhydrous methanol at room temperature for 12 h. The tissue was bleached overnight in methanol containing 7% H_2_O_2_ to decrease autofluorescence (Kikuchi and Graf, 2007) then dehydrated again in anhydrous methanol. The samples were cleared through a methanol-xylene series and embedded in paraffin. Four micrometer thick tissue sections were cut with a rotary microtome (Shandon Finesse microtome) and mounted on silane-coated glass slides. The sections were dewaxed through a xylene-ethanol series and air-dried. Sections were UV-irradiated 30 min (to decrease autofluorescence) then rehydrated through a graduated ethanol:water series before FISH analysis.

### Fluorescent *in situ* hybridization

Oligonucleotide probes used in this study were previously tested for specificity (Kikuchi and Graf, 2007) and are listed in Supplemental Table 1. Cleared slides were incubated in distilled water at 90°C for 5min, then hybridized at 55°C for 20 min in a hybridization chamber, and washed in 50 mL wash buffer at 50°C for 20 min on a rotating incubator (Tang et al., 2005). Hybridization solutions contained 25% formamide and probes at 1 μM (6–7 ng/μL). Slides were briefly rinsed with distilled water and allowed to dry before mounting with Slowfade® Gold. Negative no-probe controls were subjected to the same conditions and used to estimate autofluorescence of the samples (data not shown). The fluorescence was observed with a Nikon Eclipse T_*i*_ microscope (NIS Elements version 4.13 software) equipped with 405 nm, 488 nm, 558 nm, and 640 nm wavelength lasers and a 60× objective lens. The same excitation and emission collection settings were used for all samples. All image processing was performed using ImageJ64 software.

## Results

### Composition of the leech microbiome

**Figure 2:**
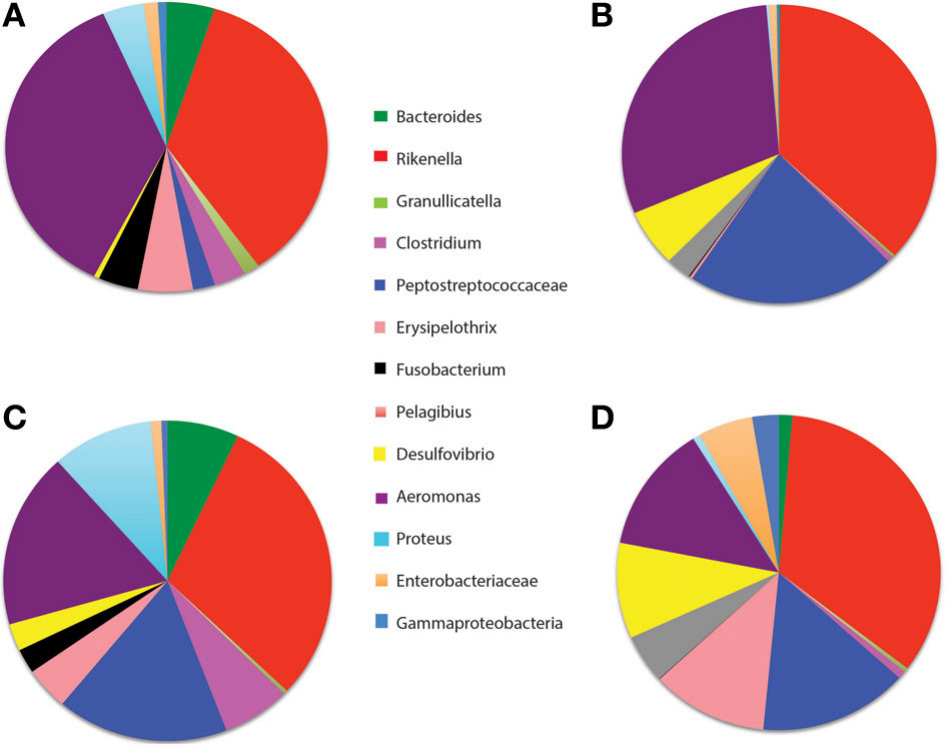
**Comparison of leech microbiome using 16S rRNA gene sequencing**. The V6 region of the 16S rRNA gene was pyrosequenced and analyzed using VAMPS. The pie charts depict the relative abundance of the 16S rRNA gene sequences, obtained from **(A)** Intraluminal fluid (ILF) of the crop harvested from animals fed one blood meal. **(B)** Intestinum contents harvested from animals fed one blood meal. **(C)** ILF harvested from animals fed two blood meals 4 weeks apart. **(D)** Intestinum contents harvested from animals fed blood two meals 4 weeks apart.

Prior studies of the leech gut microbiome relied on culturing or Sanger sequencing of clone libraries (Graf, 1999; Worthen et al., 2006; Siddall et al., 2007, 2011; Laufer et al., 2008). In this study, we utilized V6 region pyrosequencing of the 16S rRNA gene to characterize the two compartments of the leech digestive tract, the crop and intestinum, to determine the presence of less abundant organisms (Figure 1). DNA was extracted 96 h after feeding from both ILF and intestinum content. The different digestive-tract regions were harvested from four animals and equal amounts of DNA from each leech were pooled for each sample type. Sequencing the V6 region of the 16S rRNA gene confirmed that *Rikenella*-like bacteria (35% of the 16S rRNA gene sequences) and *Aeromonas* (36%) dominated the microbiome in the crop 96 h after feeding the animal one blood meal. The next most abundant sequence tags were identified to the lowest taxonomic level as *Erysipelothrix* (5%), *Bacteroides* (5%), *Proteus* (4%), *Fusobacterium* (4%), *Clostridium* (3%), Peptostreptococcaceae (2%), and *Granulicatella* (1%), which together with *Aeromonas* and the *Rikenella*-like bacteria accounted for 95% of the sequences obtained (Figure 2). *Fusobacterium* and Peptostreptococcaceae were reported previously by Worthen et al. to be present in the intestinum but were not detected in the crop (Worthen et al., 2006). Overall, these data indicate that the leech microbiome in the crop is more complex than originally reported either due to the increased sequencing depth or potentially a change in the microbiome.

In the intestinum, sequence tags from the *Rikenella*-like bacteria (36%) and *Aeromonas* (30%) indicated that these organisms also dominate this microbial community. Peptostreptococcaceae was the third most abundant taxon accounting for 21% of the 16S rRNA gene sequences. The Peptostreptococcaceae tag sequence from the 454 sequencing was 100% identical to the 16S rRNA gene clone PW11 (DQ355180.2) that was obtained previously from the intestinum of *H. verbana* (Worthen et al., 2006). PW11 was identified by the RDP Classifier as 97% identical to *Proteocatella*, which is an obligate, spore-forming anaerobe isolated from Megallanic penguin guano (Pikuta et al., 2009) and produces acetate, butyrate, and ethanol as fermentation end-products. Worthen et al. also reported *Desulfovibrio* that accounted for 6% previously in the intestinum. (Worthen et al., 2006). *Pelagibius* accounted for another 3%. These genera, together with the *Rikenella*-like bacteria and *Aeromonas*, accounted for 96% of the sequences. For a comparison of ILF and intestinum content, all datasets were normalized by randomly subsampling the data to the number of sequence reads that were obtained in the sample with the fewest reads (Maltz and Graf, 2011). The observed richness after normalization was 36 for the ILF and 60 for the intestinum sample. This increase in richness in the more distal regions of the digestive tract has been observed in other animals digestive tracts (Stearns et al., 2011).

One of our goals was to sequence the metagenome of the uncultivated microorganisms and thus we were interested in reducing the levels of *Aeromonas*. Kikuchi et al., reported different growth dynamics of *Aeromonas* and the *Rikenella*-like bacterium (Maltz and Graf, 2011). *Aeromonas* increases rapidly in abundance after a blood meal, but decreases fairly soon after 42 h. The uncultivated *Rikenella*-like bacteria in contrast, grows more slowly but persists at relatively high levels up to 96 h post feeding. To determine the best time for sampling in order to enrich the metagenome sequences for uncultivated microorganisms, we evaluated the microbial community in leeches that had received a second blood meal 4 weeks after the first. The microbiomes were similar to the animals fed only once, with the exception that in the ILF *Aeromonas* only accounted for 18% of the sequences while *Proteocatella* (17%), *Proteus* (10%), and *Clostridium* (7%) increased in abundance. The richness was similar to that observed in the animals fed only once, 33 for ILF and 62 for the intestinum. Based on these data, we chose to sequences the metagenome of the crop microbiome from animals that were fed twice and sacrificed at 96 h after feeding to favor recovery of genomic data from the less abundant microbes.

### Leech crop metagenome

The metagenome of the leech crop was pyrosequenced with the goal of gaining insight into metabolic functions of the entire community. Potential challenges with sequencing the crop metagenome included contamination with eukaryotic cells, such as leech cells or sheep leukocytes, that contain much more DNA than prokaryotic cells and that *Aeromonas* was so abundant that the other symbiont genomes received insufficient coverage. Based on the previously described population dynamics, we chose to sample 96 h after feeding the animals a second blood meal from which leukocytes had been depleted by removing the buffy coat after centrifugation of the heparinized blood. We based the timing of the two blood meals on the 16S rRNA data above. This approach allowed us to obtain a high coverage for *Rikenella*-like bacteria and obtain sequences from the less abundant organisms.

The DNA was pyrosequenced with the Titanium chemistry yielding 293 MB of sequence that was assembled into 17.4 MB of contigs greater than 500 bp. 8320 contigs were obtained with an average size of 2.0 kb; the N50 size was 2.93 kb; and the largest contig was 39,340 bp long. Over 90% of the reads were assembled (Table 1). The high percentage of assembled reads can be indicative of a metagenome with a relatively low complexity. Analysis with MG-RAST indicated that 98.3% of the contigs belonged to bacteria, 0.5% to archaea, and 1% to eukaryotes (excluding 11 contigs that matched to *Aeromonas* sequences annotated as the mosquito *Anopheles*). The *Aeromonas* sequences annotated as *Anopheles* are likely to have matched sequences obtained from the bacteria carried within the mosquitoes' digestive tract, since some species of mosquito have been shown to house *Aeromonas* species in the midgut (Pidiyar et al., 2002; Janda and Abbott, 2010). This analysis clearly showed that DNA from leech or sheep cells did not significantly contaminate our metagenome sample.

**Figure 3:**
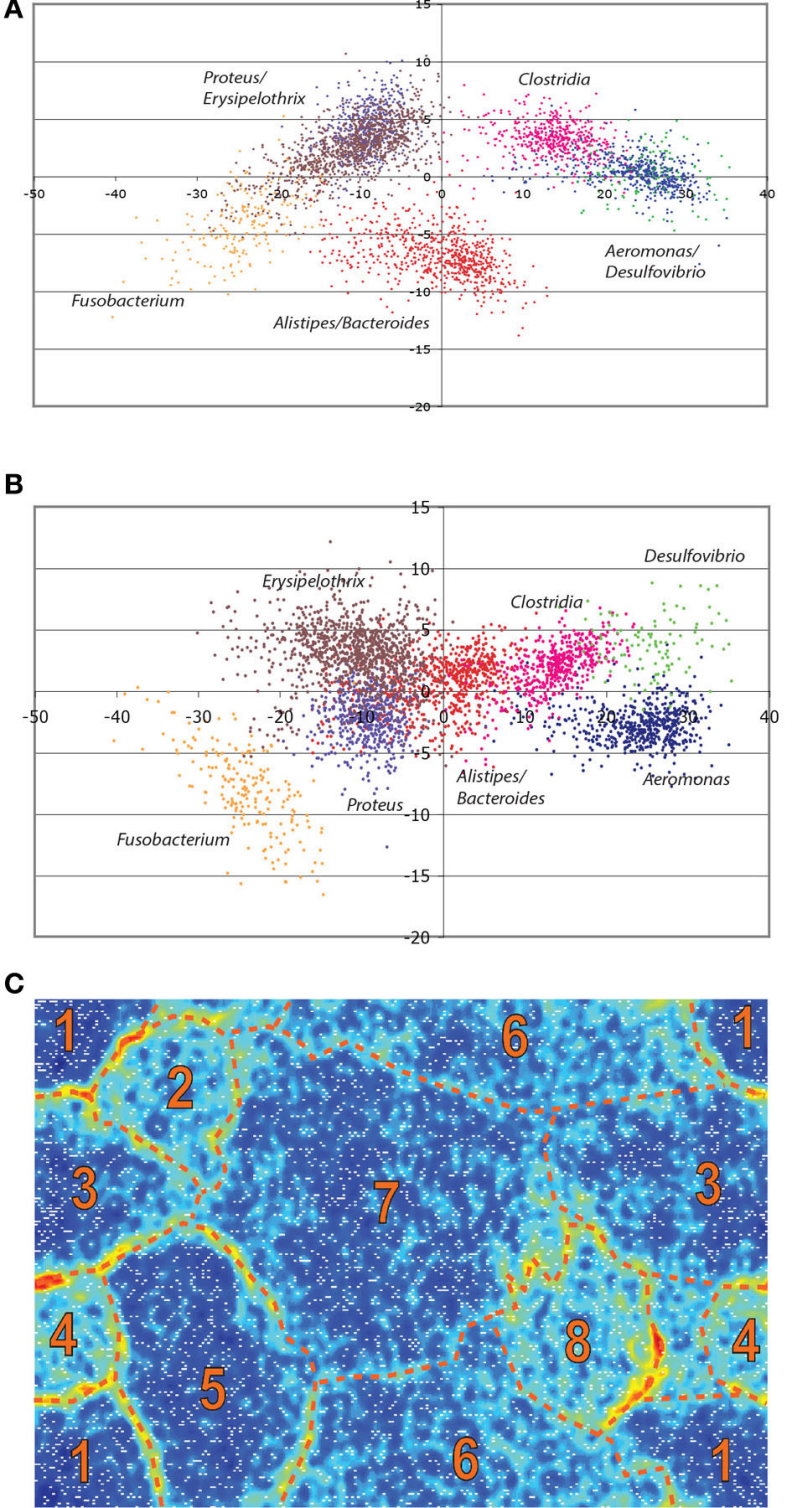
**Grouping of contigs from the leech crop metagenome into organismal bins**. We used feeding conditions that minimized *Aeromonas* to obtain greater coverage of *Rikenella* and less abundant organisms. After assembling the pyrosequenced DNA contigs were placed into organismal bins using two approaches. Each dot represent the position of a contig calculated from normalized GC percents, di-, tri-, tetra-, and pentanucleotide frequencies. The resulting PCA data consists of multi-dimensional coordinate that allowed us to separate the different members **(A,B)**. Contigs that in a dimension appears to be overlapping (*Aeromonas/Desulfovibrio* or *Proteus/Erysipelothrix*) in **(A)** can be separated using different coordinates **(B)**. Evolving self-organizing map (ESOM) of the leech crop metagenome **(C)**. Each dot represent the position on a sphere of every contig calculated from normalized tetranucleotide frequencies. The ESOM maps are composed of valleys (Blue) where contigs of similar frequencies are clustered together and separated from other clusters by ridges (Yellow/orange). In this example, eight defined clusters were found to be present in our metagenome data. Each clusters can be trace back to predominant species: (1) Aeromonas, (2) Bacteroides, (3) Alistipes/Bacteroides, (4) Desulfovibrio, (5) Clostridia, (6) Erysipelothrix, (7) Proteus, (8) Fusobacterium. The identity of the clusters was determined by comparing the contigs to the NCBI database.

Another challenge in metagenomic studies is identifying the specific organisms from which the contigs originated. We used two approaches to group the contigs, a principle component analysis of nucleotide usage patterns and GC content and evolving self-organizing mapping approach utilizing tetranucleotide usage patterns (Woyke et al., 2006; Dick et al., 2009). When selecting contigs that were larger than 1 kb, distinct clusters emerged that corresponded to individual taxonomic units and both analyses agreed for 80% of the contigs (Figure 3). BLASTX analysis of ORFs from these clusters supported this identification. Because we had chosen an experimental setup that minimized *Aeromonas* cells, we were able to recover contigs predicted to originate from *Rikenella* (3.1 MB), *Aeromonas* (4.5 MB)*, Proteus* (2.9 MB)*, Clostridium* (1.8 MB)*, Eryspelothrix* (0.96 MB)*, Desulfovibrio* (0.14 MB), and *Fusobacterium* (0.27 MB)**. It is likely that some of the contigs in the *Rikenella* bin originated from the *Bacteroides* genomes, but they would be less abundant and have less coverage. The coverage for *Rikenella* was ~20-fold, *Aeromonas ~*6–10-fold and less for the other organisms. When comparing the relative sequence coverage of contigs binned into *Rikenella* and *Aeromonas* groups, we detected insertion elements that were likely inserted at multiple locations in the chromosome of these organisms. These repetitive sequences probably led to shorter contig lengths because the shotgun assembly could not bridge the 1 kb long insertion elements.

Functional analysis was done on the leech metagenomic microbiome using MG-RAST. Analysis revealed many sequences matching genes involved in utilization of alternative nutrient sources, i.e., sialic acid metabolism, alginate metabolism, legionaminc biosynthesis, and mannose metabolism. The crop microbiome also encodes genes important for synthesis of vitamins, which are deficient in blood, including biosynthesis genes for thiamine, biotin, folate, and pyridoxine (B6) (Edward et al., 1957). The crop microbiome also encoded genes to synthesize essential branch chain amino acids (BCAA) valine, leucine, and isoleucine, as well as the ability to utilize heme.

### Comparative functional analysis of the leech-gut microbiome

**Figure 4:**
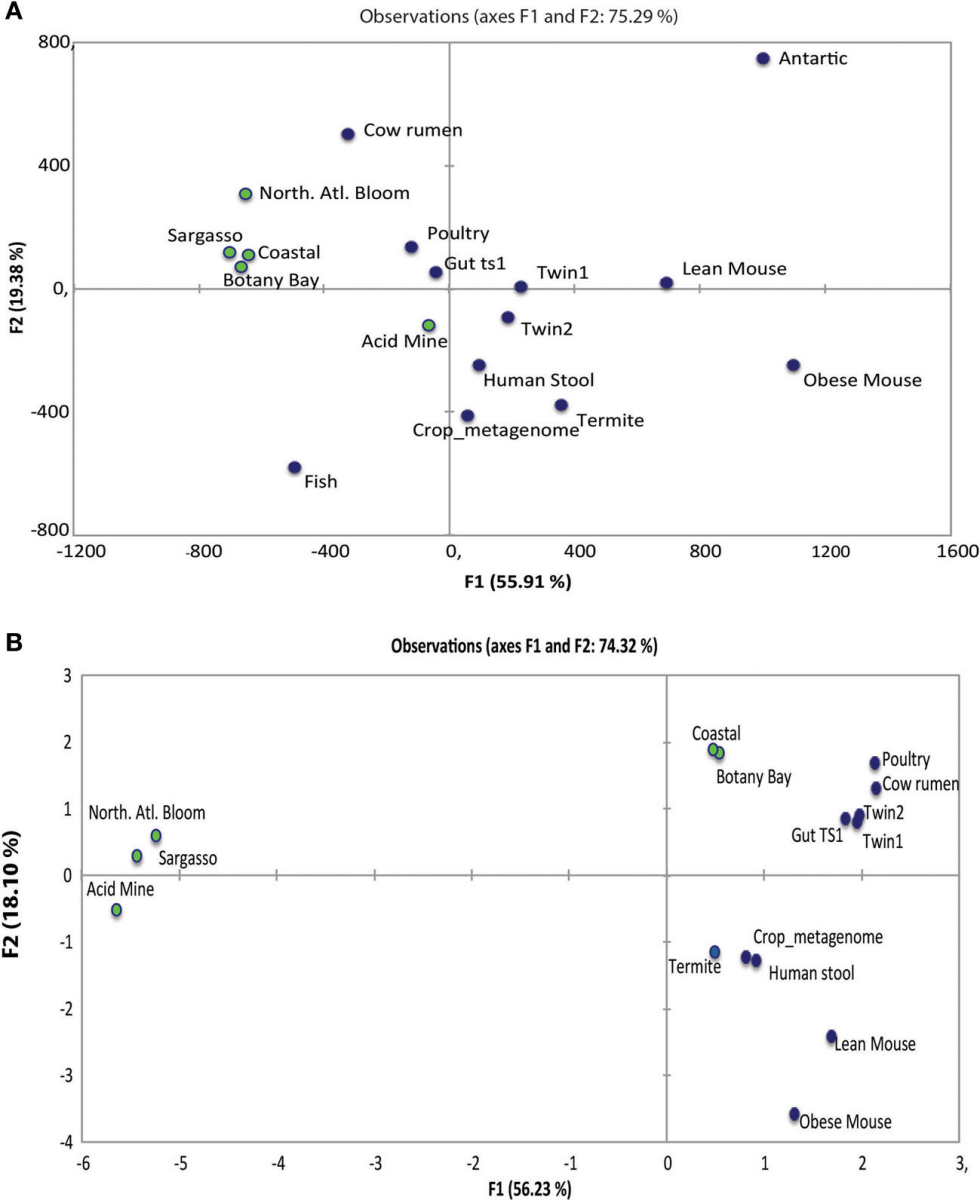
**Comparison of the metabolic functions of metagenomes**. The gene content of metagenomes from digestive-tract and aquatic samples were compared. **(A)** COG PCA plot revealing separate clustering of most gut metagenome samples and environmental metagenome samples. **(B)** KEGG PCA revealing separate clustering of most gut metagenome samples and environmental metagenome samples. Green dots represent environmental samples and blue dots represent gut samples.

One commonly raised question about model systems for the human digestive-tract microbiome is how well the model's microbiome reflects the human microbiome. We compared the metabolic potential of the leech gut microbiome to other digestive tract microbiomes to evaluate the similarity of the leech microbiome to the more complex human microbiome and to metagenomes from the aquatic environments as leeches are aquatic and could obtain microbes from their habitat. The metabolic potential of the leech metagenome was explored using COGs (Clusters of Orthologous Groups) analysis (Supplemental Table 2) (Moriya et al., 2007). COG analysis uses evolutionary relations to group functionally related genes. These data from the leech crop metagenome were compared to the COGs of human stool samples, along with samples obtained from other digestive tract models including mouse, fish, cow rumen, poultry, and termite (MG-RAST ID Supplemental Table 2). The digestive-tract samples were also compared to five different environmental samples (Figure 4A). BLAST comparison of all leech metagenome sequences yielded 14,493 hits to the COG database. From the above sequence hits, 12,090 corresponded to characterized COGs and 2403 corresponded to unique COGs.

Analysis of COG data using PCA shows that the human stool sample groups the closest to the leech crop and termite gut samples (Figure 4A). The other vertebrate gut metagenomes (poultry, fish, cow rumen, and lean mouse) and the two other human fecal samples (twin 1 and 2) had broader distribution, but still grouped together, while the obese mouse was an outlier. The environmental samples; North Atlantic Bloom (marine), Sargasso Sea (marine), Botany Bay (brackish), and Coastal bloom (marine) group together, while environmental Acid mine grouped closer to the gut samples and the Antarctic lake (fresh water) sample was an outliner. These data suggest a difference in relative abundance of genes encoding these COG categories in metagenomes obtained from environmental samples and from gut niches, including the one from the medicinal leech.

Since the *Rikenella*-like bacteria are members of the Bacteroidetes family and are found in the gut of the leech, we compared its metabolic potential to another Bacteroidetes microbe found in the human gut, *Bacteroides thetaiotaomicron*. We preformed COG analysis on the binned *Rikenella*-like sequences obtained from the leech metagenome and compared them to *B. thetaiotaomicron* (Salyers, 1984; Xu et al., 2003; Kashyap et al., 2013) (Table 2). Table 2 depicts a trend between all the COG categories suggesting the *Rikenella*-like bacterium and *B. thetaiotaomicron* have similar metabolic profiles (Kikuchi and Graf, 2007; Bomar et al., 2011). This finding suggests that these two *Bacteroidetes* gut symbionts possess similar metabolic potentials suggest that they have similar nutritional requirements.

**Table 2:** **COG Metabolic analysis of *Rikenella*-like bacterium compared with *Bacteroides thetaiotaomicron***.

	***Rikenella* blast X hits**	***Rikenell*a %**	***B. thetaiotaomicron* %**
Energy production and conversion	96	5.1	4.8
Carbohydrate transport and metabolism	112	6.0	9.8
Amino acid transport and metabolism	149	7.9	6.5
Nucleotide transport and metabolism	46	2.5	2.2
Coenzyme transport and metabolism	92	4.9	3.3
Lipid transport and metabolism	44	2.3	2.2
Inorganic ion transport and metabolism	87	4.6	6.2
Secondary metabolites biosynthesis, transport and catabolism	17	0.9	0.9

We next carried out KEGG analysis on the gut niches and the environmental niches looking for genes that differ in these two groups (Supplemental Table 2). PCA on KEGG analysis revealed that all the gut samples clustered together, leech crop and human stool clustering the closest (Figure 4B). KEGG analysis again showed the obese mouse as an outliner when compared to the other samples. The environmental samples did not all cluster together; acid mine, Sargasso, and North Atlantic bloom were clearly segregated from the other samples, indicating that the common genes found in these three regions are very different from those found in shoreline environments, Coastal water, and Botany Bay.

### Functional analysis and detection of newly identified member of the leech crop microbiome

Since *Proteus* accounted for most of the sequences after *Aeromonas* and *Rikenella* in the metagenome, we chose to further analyze *Proteus's* genes and determine its location in the leech crop. The sequences that were binned into *Proteus* group were analyzed using MG-RAST. The *Proteus* metagenome contained many central carbohydrate metabolism pathways, including glycolysis, TCA, pyruvate, glyoxylate bypass, and the Entner-Doudoroff pathway. Some specialized genes for the colonization of the leech crop could include iron acquisition genes due to the low amounts of free iron present in a blood meal. *Proteus* contains genes for the biosynthesis of a ferric iron siderophore, ferrous iron receptor, and a heme utilization mechanism. Since blood is also considered low in vitamin Bs these pathways were also examined (Edward et al., 1957). *Proteus* possesses genes for the biosynthesis of folate (vitamin B9), biotin (B7), pyridoxine (B6), and also menaquinone (K). *Proteus*, like the *Rikenella*-like bacteria and *Aeromonas*, contains sialic acid metabolism genes, fatty acid degradation, and lipopolysaccharide (LPS) synthesis pathways (Bomar and Graf, 2012; Bomar et al., 2013). Like *Aeromonas*, *Proteus* possesses several secretion pathways (Type I, II, III, and IV), catalase, outer membrane protein A and several ABC-type transporters (Rio et al., 2007; Silver and Graf, 2009; Bomar et al., 2013).

**Figure 5:**
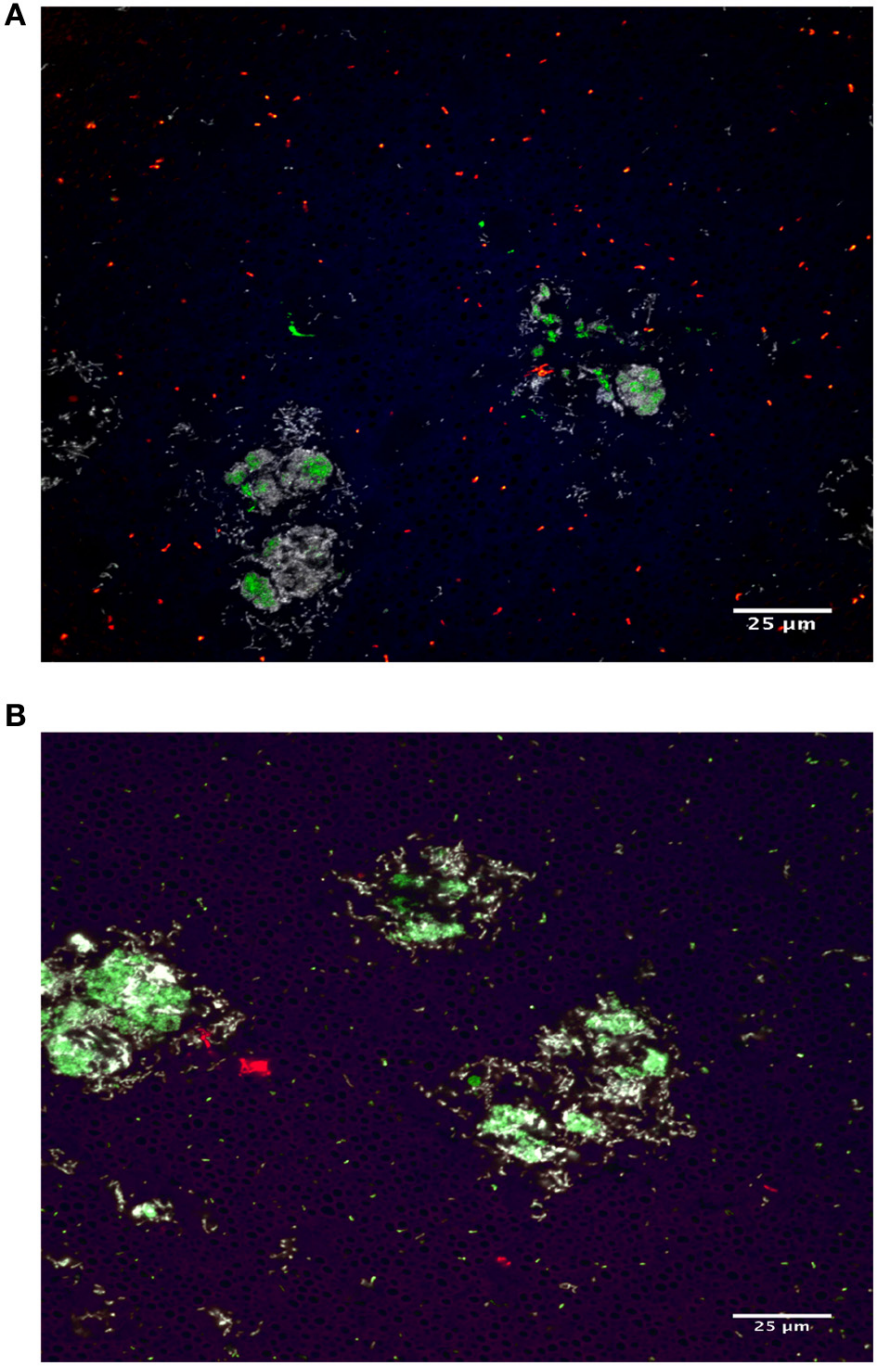
***Proteus* resides in microcolonies inside the leech crop**. The location of bacteria within the crop was determined using fluorescence *in situ* hybridization. Fixed sections of the leech crop were stained with: **(A)** Eubacterial (EUB338, green), Bacteroidetes (CF319a, white) and *Aeromonas* (AER66, red); **(B)** Eubacterial (EUB338, green), Bacteroidetes (CF319a, white), and *Proteus* (PRO1A, red). Tissue autofluorescence can be seen in blue/purple. Dark areas are suggestive of polysaccharide layers surrounding bacterial aggregates. For detailed probe information, see Supplemental Table 1.

Understanding the location of microorganisms can lead to predictions of interactions with other microbes or the host, and may provide clues for determining the nutrient available to the microbes i.e., erythrocytes vs. mucin. We determined the microniche that *Proteus* inhabits using FISH with universal 16S rRNA, *Aeromonas*, Bacteroidetes (*Rikenella*-like), and *Proteus* probes (Figure 5). These data confirmed the formation of microcolonies within the leech crop ILF (Bomar and Graf, 2012) and revealed that *Proteus* is also present within the microcolonies (Sanguin et al., 2006, Figure 5B). Thus, the composition and inter-species interactions within the microcolonies appears to be more complex than previously reported.

## Discussion

In this study, we used 16S rRNA gene tag surveys and metagenome sequencing and discovered a more complex composition of the leech digestive-tract microbiome than has previously been known. Although the relative abundance of different taxa is affected by both copy number of the ribosomal operons and the amplification efficiency, it has been shown to be a powerful tool in examining composition in many systems (Dethlefsen et al., 2008; McFall-Ngai, 2008a,b; Turnbaugh and Gordon, 2009; Turnbaugh et al., 2009; Roeselers et al., 2011; Wong et al., 2011; Nyholm and Graf, 2012; Wong et al., 2013). This 16S rRNA tag survey also confirmed the dominance of two taxa, the *Rikenella*-like bacterium and *Aeromonas*. This consistent detection of *Aeromonas veronii* and the *Rikenella*-like bacterium suggests that these two species comprise the core symbionts of the leech (Worthen et al., 2006; Kikuchi and Graf, 2007; Nelson and Graf, 2012). These animals were obtained from a commercial leech supplier, which markets to hospitals for the use on patients receiving leech therapy. *Aeromonas* is also a human pathogen and can cause infections in patients receiving leech therapy (Whitlock et al., 1983; Lineaweaver et al., 1992; Whitaker et al., 2011) thus sellers attempt to reduce the *Aeromonas* population in an effort to reduce wound infections and septicemia. It remains to be determined whether the changes in complexity are due to changes in animal husbandry, different geographical origin, or improved technology. More comprehensive surveys of animals from different leech farms as well as field caught animals are needed to determine which of the additional organisms identified belong to the core microbiome, to a transient population or a transient population that persists in the gut or organism due to a disturbed niche. One could extend the concept of resident and tourist organisms (Savage, 1977; Dunn and Stabb, 2004) by adding another category, squatters, for organisms that move into niche that was previously occupied by other organisms (Darby and Scott, 2007). We are currently comparing the composition of the microbiome of individual animals obtained from different sources to confirm the presence of this less abundant organism to evaluate if they are members of the core microbiome. In addition, we are attempting to culture these newly identified microbes to explore the role of these organisms in the leech digestive tract (Bomar et al., 2011).

Metagenomic analysis of the leech crop provided insight into gene content and possible metabolic functions that the leech gut symbionts possess and might contribute to the host. Within the metagenome of crop, genes were found that are likely important for survival of the symbiont and contribution to the host. Because blood is poor in vitamins (Edward et al., 1957), microbial production of vitamins is likely to be critical for leech health. Insects with restrictive diets often possess symbionts that produce essential amino acids and/or vitamins missing from the host diet (Friend, 1958; Dadd, 1961; Schowen, 1998; Snyder et al., 2012). Blood feeding insects, such as mosquitos and bed bugs, harbor symbionts that produce essential vitamins. Furthermore, the ability of the microbial gut members to acquire iron from heme is suggested by the presence of heme utilization genes and likely important for the proliferation in the leech gut since blood is low in free iron. Iron acquisition using siderophores and heme receptors is often considered an adaptation to the host environment since iron is usually bound in heme groups within the host (Graça-Souza et al., 2006). Future studies on the iron acquisition systems would provide insight into the expression and function of these genes. Having genetic tools available for *A. veronii*, we can directly test this hypothesis (Aggrawal and Silverman, 2007; Adin et al., 2009; Bomar et al., 2011). This functional analysis does provide reinforcement to previous hypotheses that the leech gut inhabitants have the potential to provide essential nutritional needs to the host, which has been hypothesized in many digestive tract symbiosis including humans.

Model systems have been widely used in biology to further our understanding of animal development and the immune system (Nyholm and Graf, 2012). The complexity and variability of the human microbiome calls for model organisms which are experimentally amenable and less variable while still providing relevant insight for vertebrate biology (Ruby, 2008). Using COG and KEGG analysis we tried to address if the leech gut microbiome reflected the metabolic potential of other animal digestive systems. COG data indicated that the leech crop, human stool, and termite gut samples cluster together, revealing the presence of a common set of proteins that are important for these gut-associated niches. COG results also showed that the marine environmental samples clustered together indicating a core set of genes for these metagenomes. The other vertebrate gut metagenomes (fish, cow rumen, poultry, twin 1, twin 2, and lean mice) seemed to be more dispersed from the other gut communities, but still loosely clustering together. KEGG analysis grouped all gut samples together, human stool sample and the leech crop clustering the closest. COG and KEGG differences could be due to the differences in the databases. The COG database is generated by comparing the protein sequences of complete genomes while; the KEGG database is generated by genomes and information about biochemical compounds and reactions. Interestingly, in both the COG and KEGG analysis the obese mouse metagenome was an outlier when compared to the other gut samples. It has already been shown that the microbial community of obese humans not only differs from lean humans but also has the ability to harvest more calories from food (Turnbaugh et al., 2006). These data reinforce just how different the metabolic potential could be at this diseased state from other gut communities (Backhed et al., 2005). Together these data suggest that there is a core group of genes that are conserved among diverse digestive-tract microbiomes, including in animals feeding on highly restricted diets such as wood and blood, suggesting the appropriateness of the leech as a model for digestive-tract symbioses in general. Interestingly, when human microbiome samples were analyzed for the species composition and functional content, it was found that even when species composition varied greatly, the functional capabilities were conserved (Human Microbiome Project, 2012b). This extends the functional conservation of the microbiome across a diverse range of animals ranging from invertebrates to mammals. It will be interesting to perform a broader and more detailed analysis of the metagenomes of a wide range of animals across a range of taxa and nutritional preferences.

We also found a similar gene content for the *Rikenella*-like bacteria and the human gut microbe *Bacteroides thetaiotaomicron*. COG analysis on the *Rikenella*-like sequences obtained from the leech metagenome and *B. thetaiotaomicron* revealed that even though these two species of bacteria are harbored within animals that have very different diets, their genomes encode for proteins performing similar functions. We previously hypothesized that the *Rikenella*-like bacteria, like *B*. *thetaiotaomicron*, could be feeding on glycans of glycosylated proteins such as host produced mucin (Bomar et al., 2011). This could explain why the *Rikenella*-like bacteria are able to sustain higher populations densities for a longer period of time after feeding, while we predict that *Aeromonas* runs out of readily accessible nutrients, as suggested by the population decline by 1 week after feeding (Kikuchi and Graf, 2007; Bomar et al., 2011).

Since *Proteus* was the third most abundant microbe in the metagenome, we chose to analyze *Proteus* for adaption to life within the leech crop. The *Proteus* sequences were all very similar to what would be expected of a Gram-negative bacterium. It did however possess genes that have been shown or could be important for leech crop colonization. Sialic acid metabolism has been shown to be present in HM21 (an *A. veronii* isolate from the medicinal leech) draft genome (Bomar et al., 2013), indicating that *Proteus* could have the ability to forage on sialated glycoproteins on the surface of ingested erythrocytes or on host mucin glycans (Bomar and Graf, 2012). Like *Aeromonas*, *Proteus* sequences encoded several potential adaption factors i.e., LPS assembly pathways, several secretion pathways, catalase, iron acquisition genes, and outer membrane protein A. The type three-secretion system (T3SS-1) and LPS have been shown to be required for *Aeromonas* colonization of the leech gut (Braschler et al., 2003; Aggrawal and Silverman, 2007; Silver et al., 2007). LPS and outer membrane protein A has been shown to be important for evading the host immune system in other symbiosis; i.e., Hawaiian bobtailed-squid and tsetse flies (Weiss et al., 2008; Maltz et al., 2012; Nyholm and Graf, 2012). The predicted genome content indicates that *Proteus* possess the ability to obtain nutrients. The metagenome data suggests that *Proteus* can biosynthesis folate, biotin, and pyridoxine thus potentially providing the host an important vitamin B that blood lacks. For example, folate is a water-soluble B9 vitamin that is a coenzyme in single-carbon transfers in the synthesis of nucleic acids and metabolism of amino acids. Symbiotically synthesized folate has been shown to be important for fitness and development in *Drosophila melanogaster* (Blatch et al., 2010).

Finally, we were able to visualize one of the newly identified members of the leech microbiome (*Proteus)* through FISH. *Proteus* are part of the Enterobacteriaceae family and are commonly found in the environment and in the normal flora of intestinal tracts of mammals, birds, and reptiles (O'Hara et al., 2000). We detected *Proteus* signal within the microcolonies in the leech crop but it remains to be shown that this species or any of the newly identified microbes are members of the core microbiome. Future studies will explore these microbe–microbe interactions and will also investigate how these gastrointestinal bacteria affect their host.

These data as a whole reinforce previous studies showing the presence of two culturable, dominant species in a moderately complex digestive-tract microbiome and have provided further evidence that the medicinal leech is a simple and relevant model system for studying digestive-tract symbioses. Future 16S rRNA gene surveys of the leech crop microbiome will investigate the stability of the microbiome over time and during perturbations. These data also gave insight into potential functions of the leech crop microbiome. The availability of a metagenome that can be supplemented by genomes from specific cultured strains will greatly enhance our ability to perform metatranscriptomic studies, which provide insight into the *in vivo* physiology of the microbiome.

### Conflict of interest statement

The authors declare that the research was conducted in the absence of any commercial or financial relationships that could be construed as a potential conflict of interest.
