# Supplementary material for: Metagenomic analysis of the medicinal leech gut microbiota
Source: Front Microbiol. 2014 Apr 17;5:151. doi: 10.3389/fmicb.2014.00151 (PMC4029005; doi:10.3389/fmicb.2014.00151)
Supplement: Supplementary file 1 [file DataSheet1.PDF]

Supplemental Table 1: FISH probes used in this study

Probe Name	Organism	Fluorophore	Probe Base[6] Accession #	Sequence
**Eub338**	*most Bacteria*	Alexa 488	pB-00159	[AminoC6+Alexa488]GCTGCCTCCCGTAGGAGT
**CF319a**	*Bacteroidetes*	Cy5	pB-00042	[Cy5]TGGTCCGTGTCTCAGTAC
**Aer66**	*most Aeromonas*	Cy3	n/a	[Cy3]CTACTTTCCCGCTGCCGC
**Pro1A**	*Proteus*	Cy3.5	pB-02110	[Cy35]ATGGGTTCATCCGATAGTGC

Supplemental Table 2: Metagenome MG-RAST ID numbers for COG and KEGG Analysis

**Name of Metagenome**	MG-RAST ID Number
**Gut Samples:**	
Twin Gut 1	4440452.7
Twin Gut 2	4440613.3
Human Stool	4444130.3
Fish Gut	4441695.3
Termite Gut	4442701.3
Cow Rumen (pooled planktonic)	4441682.3
Poultry Gut	4444843.3
Gut ts1	4440452.7
Lean mouse gut	4440463.3
Obese mouse gut	4440464.3
**Environmental samples:**	
North Atlantic spring bloom	4443732.3
Antarctic aquatic 1 lake	4443683.3
Botany Bay Australia	4443688.3
Coastal: Cape May NJ US	4441144.3
Sargasso Sea	4443727.3
Acid mine	4441137.3
